# Hospital and Institutional News

**Published:** 1915-12-25

**Authors:** 


### HOSPITAL AND INSTITUTIONAL NEWS.

### VISIT OF THE QUEEN TO FULHAM MILITARY HOSPITAL.

HER MAJESTY THE QUEEN paid a surprise visit to the Fulham Military Hospital on Wednesday, December 15. She was attended by Lady Mary Trefusis, and was received by the Commandant, Major C. Thackray Parsons. Several members of the medical staff and of the Board of Guardians were in attendance, and, with the heads of the nursing staff, were presented to Her Majesty, who then visited seven of the wards, in which a number of the patients had been collected. The Queen spoke to each of the patients in these wards, and was particularly interested in the men of the Colonial contingents. She also inspected the operating unit, the *x*-ray room, and the massage and electro-therapeutic department. Dr. Stoney, the radiographer to the hospital, exhibited a number of skiagrams of patients in the wards, and also demonstrated the high-frequency apparatus and the electrical vibrator. It happened to be visiting day at the hospital, but, upon being informed, the Queen, with her usual kindness, gave instructions that the visitors should not be stopped. At her departure she was heartily cheered by a large crowd, which had quickly collected outside the hospital.

### KING EDWARD VII.'S HOSPITAL FUND.

THE King's Hospital Fund has occupied considerable space in our columns during recent weeks, a fact which emphasises its ever-growing importance. The report we give elsewhere of the meeting of governors at St. James's Palace on the 16th instant is both interesting and important. It is a remarkable fact that in nineteen years this Fund has distributed upwards of £2,000,000, whilst its invested funds represent at least £2,000,000 more. The continuous interest of H.M. King George in this Fund is shown by his letter. We hope that every one of his subjects throughout the Kingdom and Empire realises the extraordinary value of his personal service for the sick and of the interest displayed in all philanthropic objects by Queen Mary. The Speaker's speech at the King's Fund meeting should be weighed and read. It is statesmanlike and sound. It has special interest from the misfortune of his compulsory absence from the League of Mercy meeting owing to the urgency of public affairs. A remarkable feature of the meeting was Sir Ernest Cassel's renewed and splendid generosity to the hospitals. Sir Ernest's action has a value far beyond the large sum involved. It testifies to and confirms the soundness of the basis for their gifts adopted by wealthy givers referred to in our Christmas Number. It is a wise practice to base their gifts upon accurate data and actual results.

### THE METROPOLITAN HOSPITAL SUNDAY FUND.

WAR conditions, owing to the wise and energetic direction of the vice-chairman, Sir C. Ernest Tritton, whole-heartedly supported by an excellent staff, and the vigorous and united efforts of the clergy, have not adversely affected the finances of this Fund. It is, therefore, most encouraging to be able to record that London, like most of our great cities, displays this year an awakened and extended interest in Hospital Sunday. Canon Miller's organisation was the first great fund to awaken and quicken free offerings to voluntary hospitals from a free people. The following remarkable facts of the year are welcome and should be studied. The amount available for distribution to the hospitals has been increased by £12,629. The contributing congregations in 1915, the 43rd year of the Sunday Fund in London, numbered 2,186. The actual amount contributed by churches and congregations shows an increase of £10,487; and the percentage of expenses of working the Fund are one-half per cent. less than in the previous year, and also than the average yearly working expenses during forty-three years. Nothing could more convincingly demonstrate the splendid results achieved under the able administration of Sir C. Ernest Tritton and the secretaries, Mr. Arnold James and Mr. Lander. They will receive, and have well won, the admiration and acknowledgment of all hospital workers.

### THE LEAGUE OF MERCY.

IT is gratifying to find that the impetus given to this organisation by the New Year's letters of the late treasurer in the *Times*, backed by the energetic and untiring efforts of the new secretary, Mr. A. H. Franklyn, together with the special efforts of the presidents and lady presidents, vice-presidents, and honorary secretaries, especially in the districts mentioned by Sir Frederick Green on page 286 of this issue, have produced a total yield of £19,582. We are particularly pleased to note that our forecast of the possibilities has been eloquently justified by the grand work done by Lady Kilmorey in the Westminster district. Westminster was the first real starting-point for the progressive development of the League in the early days, thanks to Mrs. Corkran, the then president. Lady Kilmorey has done a splendid work, and she has well won the proud position of being president of the first district on the League's list this year. The president and all present at St. James's Palace on the 21st instant were interested and helped by Lord Sandhurst's frank and interesting address (see p. 277). We welcome Lord Sandhurst back to the voluntary hospitals field and congratulate him on his well-earned encouragement by a spontaneous gift of £1,000 to St. Bartholomew's Hospital from Sir Ernest Cassel (p. 284).

### THE SATURDAY FUND AND THE HOSPITAL SITUATION.

WE dealt last week, under the heading "Patriotism and Personal Service," with the growth of public generosity, and can now illustrate this by some figures given in the *Hospital Saturday Fund Journal*. "At the time of writing," says our contemporary, "the Fund's receipts exceed those for the corresponding period of 1914 by £4,000. If contributions continue to flow in until January 10, when the accounts for 1915 are to be closed, as they have done during the year, the zenith reached in 1911, before the set-back caused by the operations of the National Insurance Act, may be reached, or even surpassed.'' That is a very wonderful statement. It is the justification of the voluntary system, for it shows how deep is its hold upon the nation. There is another aspect of the Saturday Fund's work which equally deserves to be recorded. The Fund is the normal channel through which complaints of delay in treating patients are received, but, notwithstanding the hospitals' decision to make the sick and wounded soldier their first care during the war, the number of complaints received, we are told, have been very few. In regard to the supply of doctors —a matter on which the direction, be it noted, has come from above and not, as in the case of public generosity and public patience, from below—the Journal naturally records the serious misgivings caused by the recent encouragement of medical students in their first, second, and third year to enlist. Dealing with this situation in the light of Lord Derby's scheme, the proposal is recorded that in London all medical men and dental students connected with the medical schools should be placed, regardless of age, in a specially late group. This group could then be subdivided into five classes, according to the length each man's studies had proceeded. "All students," we read, "should be entitled and directed to return to their work at the hospitals, receiving no Army pay, but wearing uniform. Those who did not take commissions should receive preference for the R.A.M.C., in which their previous training should be of value. Those who were medically qualified should take commissions." Considering the class of patient which comes under the eye of the Hospital Saturday Fund, the situation that would be created by a shortage of doctors is fully realised; and, since the unwarrantable belief that the war will soon be over could alone excuse the absence of any provision for the future, it is only statesmanship to take measures to secure it. But the English nation, which shines in voluntary organisation, has the defect of an absence of this virtue in the business of government.

### THE RED CROSS BUDGET.

A most valuable and interesting report of the Joint Finance Committee of the British Red Cross Society and the Order of St. John of Jerusalem has just been submitted to the Joint War Committee of those bodies, dealing with the year ended October 20 last. Into the details of this important document, which comprises eighty-five pages, we hope to be able to enter at greater length on a future occasion; for the moment, it suffices to quote merely some selected items which illustrate the enormous revenue and expenditure handled by the Joint Committee. The income for the year was more than a million and three-quarters (£1,864,036), and all but two hundred and twenty thousand of this vast sum was expended. The management expenses at home and in every theatre of war, excluding hospitals, were seventy-three thousand pounds, or, roughly, 4 per cent. of the expenditure. Hospital stores and comforts to the value of £463,455 were distributed, at a cost of less than 3 per cent. for handling. Transport of wounded, mainly for motor ambulances, absorbed £615,963. These two are the principal items. Then in France and Flanders (excluding motor ambulances) there has been spent £293,509; in Malta and the Near East, chiefly at Gallipoli, £194,812; in Serbia and Montenegro, £43,846; on hospital trains, £48,097; on the British Red Cross Hospital, Netley, £69,758; on the St. John Hospital, Etaples, £42,818; on the King George Hospital, Stamford Street, London, £49,153; on the Calais Enteric Hospital, £17,643; on hospitals and convalescent homes in Egypt, £25,604; and on the Australian Forces in Egypt, £21,054. Truly, a wonderful budget.

### BESTOWAL OF RED CROSS ORDERS.

A new batch of promotions and appointments to the Order of St. John of Jerusalem (in England) has been sanctioned by the King. We note with satisfaction that a larger proportion than usual of medical men have received recognition, and that there are fewer awards of a purely "Society" character. Six medical men are appointed Knights of Grace and two to be Esquires. Ten new Ladies of Grace are also in the list. Furthermore, the Order of Mercy has been bestowed on a number of workers for services rendered to the League of Mercy. A President, four Vice-Presidents, a long list of lady Vice-Presidents, and a number of members of both sexes have received the Order. Incidentally, it is worthy of note that the League has collected £600 more than last year, and has distributed, as usual, £14,000 to King Edward's Hospital Fund and £2,878 to various voluntary hospitals, an increase of £522 over last year.

### GRUMBLINGS IN THE PUBLIC HEALTH SERVICE.

A correspondent of the *Medical Officer* is highly indignant, and his sentiments are editorially endorsed, over the increasing labour of a purely clerical kind exacted from medical officers of health by recent enactments; and especially over the provisions of the recent Order of the Local Government Board making measles and German measles compulsorily notifiable diseases. What with infectious disease registers, tuberculosis registers, ophthalmia neonatorum registers, and births registers, a great part of the working day, the editor remarks, is spent in making entries, and the M.O.H. is prevented from going about his district and attending to his important duties. It is the exception, it is added, for any allowance to be made for travelling or other expenses incurred through new duties imposed by recent Orders and regulations; and it is suggested that the Local Government Board should insert clauses in future Orders requiring local authorities to provide their medical officers with adequate travelling expenses and clerical assistance. At present many medical officers of health have to provide offices, stationery, clerical assistance, and travelling expenses out of their salaries; this arrangement Mr. Herbert Samuel was credited with the intention to reform, but he left the Local Government Board for another Department and nothing was done in the matter.

### CHRISTMAS IN THE WARDS.

Last year there was some doubt in the minds of hospital men as to the best way in which Christmas should be kept. Christmas in war was an unpleasant novelty; there was a new type of patient to be considered; the staff was shorter and barely used to the new demands upon it; and there was, with all this, the latent feeling that somehow or other Christmas must be kept. In the end practical difficulties rather than anything else determined what modifications were made. Entertainments, on the lavish scale sometimes indulged in at this season, were curtailed—there was too much work to be done to organise them; decorations were less elaborate, but on the whole the alterations were in degree merely. This year, if anything, Christmas is being kept in more of the old spirit, for war work is becoming a stern habit with many of us, and the strain, though great, is one to which we have grown accustomed. The nation has been thoroughly aroused; it is unanimous and determined, and this growing spirit of solidarity, the feeling of the race and the Empire as one family, will help the staffs and the patients alike, in spite of their trials, to keep the feast in that spirit of joy which is the reward of strength and makes a hospital ward at Christmas time one of the best and most cheerful places in which to spend it. The gramophone loses little of its popularity, while it is improving its manners, and there are few patients who are otherwise than pleased with its Christmas greetings!

### THE WOUNDED AND THE BOOK MARKET.

One of the by-products of the war, which should have a suggestion for hospital administrators who take a pride in having every detail up to date, has been an enormous increase in the already big demand for the cheap book and the pocket edition. The result has been that many a series lying on the remaindered market has been bought up and in some cases even reprinted, and the cheap translation, which has never been very popular in this country, is beginning to enjoy the popularity that the present comparatively high standard of translation deserves. The book for the pocket, which is also the cheap book, is now enjoying a practically unlimited demand, and our wounded are having a good as well as a handy range of literature to choose from. We mention these facts because they remind us of the dilapidated and miserable condition into which most hospital libraries for patients have usually fallen, and suggest that the advent of the soldier into the wards of a voluntary hospital, who has brought many minor changes with him, may also leave an improved patients' library behind him when he has left. After forty years of popular education the nation has begun to read, and the time has come for every hospital to have on its shelves for the use of patients something more attractive, within as well as without, than a parcel of stale and dog-eared magazines.

### V.A.D. GATHERING AT LEICESTER.

In the Special Number of The Hospital devoted to questions of transport, which appeared earlier in the year, Mr. A. W. Faire, County Director of the V.A.D., Leicester, gave some account of the work which had been done in that city to secure that the last link in the chain of transport from the field to the base hospital at home might be completed satisfactorily. A special gathering of these members of the town V.A.D., together with the drivers of the private cars and their wives, lately took place, which was intended to be some little acknowledgment of all they had done in detraining a total of sixty-eight convoys. Among those present was Major McAllister Hewlings, who was mentioned by Mr. Faire as being the first medical man to attend the King after his recent accident in France. In looking back on the work accomplished since the outbreak of war, and on the difficulties encountered by the public apathy which prevailed beforehand, Mr. Faire said that he had then predicted that people would be tumbling over one another to do the work if war occurred. During the progress of the entertainment, which concluded with a concert, a convoy arrived, and sixty men in the transport service and sixteen women members had to leave to meet it—a fact which typ[illegible] the varied hours and times when their services have been required. It was a good idea of Mr. Faire's to organise the gathering, since this work requires much unostentatious self-sacrifice, and has been quietly and efficiently done.

### THE CASE OF OUR MERCHANT SEAMEN.

It is sometimes said that Great Britain is the only maritime Power without regular provision for its merchant seamen, and this deficiency is still sufficiently glaring to command attention and assistance for the work performed by the Royal Alfred Aged Merchant Seamen's Institution, which provides a home or a pension for many of the aged and otherwise destitute men of our Mercantile Marine. It is nearly fifty years since the Royal Alfred was established, and the Home at Belvedere has done much for a peculiarly unostentatious, but brave and cheery class of men, whose normal life is sufficiently full of hardships and peril to have more than earned adequate provision for their last decade or so. Indeed, we have only to remember the way in which the British Mercantile Marine has supplemented the work of the Navy in such hazardous tasks as mine-sweeping, and the bravery with which its men have faced the submarine peril, to realise that the gratitude of the nation cannot be too sincere to our merchant seamen. The Royal Alfred Seamen's Institution has claims just now that are really unique. Why, then, is it that these claims are not in everybody's mouth? The authorities of the institution have a great responsibility, for there is no season for them like the present, and if their institution is not caught by the floodtide of public generosity which is bearing other welladministered institutions into financially smooth waters, the supporters of the Royal Alfred Institution will have grave cause to complain. Mr. J. Bailey Walker, the secretary, and his committee are the trustees of posterity in this matter. The present opportunity must not be allowed to slip by.

### CIVILIAN DOCTORS AND THE R.A.M.C.

Some few weeks ago (The Hospital, October 2, p. 5) attention was very pointedly drawn in these pages to the lack of system whereby civilians temporarily commissioned in the Royal Army Medical Corps have not been and are not being employed to the best advantage. The remarks in question reflected accurately the feelings, often strongly expressed by, and practically unanimous among, those with actual experience in the British Expeditionary Force; and there has been ever since in this country a steadily simmering discontent with the administration of the R.A.M.C. in France. At last this has become vocal in the columns of the *Daily Telegraph*, and the correspondence in that newspaper must have awakened Sir Alfred Keogh to some of the reasons why the results of his recruiting campaign have not quite come up to expectations. We can testify that those who have written to the *Telegraph* are not merely "grousers" or men with fancied grievances; indeed, their disclosures understate, if anything, the conditions which have caused so much discontent and so many resignations. The instances quoted of overlapping and waste of medical officers are not mere isolated and occasional exceptions; they are quite common, and are the result of want of forethought and of capable supervision from above (in France, that is, not at the War Office).

### AN ADDITIONAL INSTANCE.

One of the grossest examples of mismanagement quoted by the *Telegraph's* correspondents is that of a London hospital surgeon who found himself employed as medical officer to an infantry battalion, where his work consisted of applying first-aid bandages and transferring the wounded as quickly as possible to a hospital, where they were operated on by a newly-qualified man, until recently his own dresser! We can cap that story by one related to us last week by an officer fresh from the Front. In one division there are (or were recently) three Fellows of the Royal College of Surgeons doing duty as regimental medical officers in the trenches. We can also corroborate the contention that at base hospitals there have been most unnecessarily large staffs and far too frequent changes among them; but our information goes to show that both these evils have been less extensive lately, probably owing to the difficulty of getting recruits. It seems as if the only way to bring these facts home to the War Office authorities is by the bitter experience of shortage in the supply. Thus, and thus only, can they be induced to open their eyes to a state of things which has long been common knowledge—namely, that the medical profession is gravely dissatisfied with the management of the R.A.M.C. in France.

### THE ADVISORY BOARD FOR MEDICAL SERVICES.

Nor is the *Daily Telegraph* the only important organ of public opinion that is criticising the Army Medical Service. A "Hospital Surgeon" writes to the *Morning Post* to point out that the Advisory Board for Medical Services has not met since the war began. This is the more astounding in that the members of it (including four civilians) still appear as such in the Army List, and still draw salaries of £200 a year each for it! The Board was constituted as a result of the experience of the South African War, and the civilian section of it should surely have been the most competent authority to advise the War Office on the problems of the supply of civilian doctors for the Army; indeed, it was partly for that very purpose that civilians were placed upon the Board. The *Morning Post's* correspondent roundly alleges that it is the Director-General of the Army Medical Service who has thus suppressed the Board by the simple process of never summoning it; and he blames the autocratic control exercised by the Director-General for the blundering way in which the medical student question has been handled. We know well the very great services which Sir Alfred Keogh has rendered to the country and the vast amount of work which he undertakes. But there would seem to be something in "Hospital Surgeon's" contention that he has more work than one man can possibly do, and that he should delegate parts of it to the Advisory Board, which at present draws salaries for doing nothing.

### LARGE REVERSIONS FOR INCURABLES' HOSPITALS.

One of the most notable "institutional" wills of the week is that of Miss Catherine Elizabeth Harrison, of Hampstead Lane, Highgate, who died on October 14. Subject to the life interest of her sister, and to some legacies to relatives and others, she bequeathed the ultimate residue of her estate, which will amount to about £9,000, to be equally divided between the Royal Hospital for Incurables, Putney, and the British Home for Incurables at Streatham. In each case the substantial sum thus received by these institutions is to be known as the "In Memoriam Ann Harrison" Bequest.

### THIS WEEK'S DRUG MARKET.

The most notable event that has happened since our last report appeared is the fixing, by the makers, of the prices of bromides. For some time past the position has been very perplexing because makers have had no official quotations; as might have been expected in view of the inadequacy of supplies, the new prices are very high, being more than twenty times those ruling just before the war. It seems most probable that the high rates will be maintained. Ipecacuanha is again dearer, and still higher prices are expected as there is such a large demand for this raw material for the purpose of the manufacture of emetine. In synthetic drugs there have been few further changes in price, but the extreme values which are quoted for practically all of them are well maintained. Eucalyptus oil is dearer and in good demand.

